# Daily Cigarette Abstinence and Smoking Rate With Varenicline: Relationships With Treatment, Craving, and Affect During the First Week of the Quit Attempt

**DOI:** 10.1093/ntr/ntaf095

**Published:** 2025-05-04

**Authors:** Sarah S Tonkin, Jennifer M Betts, Ashley N Dowd, Martin C Mahoney, Paul M Cinciripini, Robert A Schnoll, Tony P George, Rachel F Tyndale, Larry W Hawk Jr.

**Affiliations:** Health Promotion Research Center, University of Oklahoma Health Sciences Center, Oklahoma City, OK, USA; William S. Middleton Memorial Veterans Hospital, Madison, WI, USA; Center for Tobacco Research and Intervention, University of Wisconsin School of Medicine and Public Health, Madison, WI, USA; Addiction Sciences Division, Department of Psychiatry and Behavioral Sciences, Medical University of South Carolina, Charleson, SC, USA; Department of Internal Medicine, Roswell Park Comprehensive Cancer Center, Buffalo, NY, USA; Department of Behavioral Science, UT MD Anderson Cancer Center, Houston, TX, USA; Department of Psychiatry, University of Pennsylvania, Philadelphia, PA, USA; Temerty Faculty of Medicine, University of Toronto, Toronto, ON, Canada; Centre for Addiction and Mental Health (CAMH), Toronto, ON, Canada; Temerty Faculty of Medicine, University of Toronto, Toronto, ON, Canada; Centre for Addiction and Mental Health (CAMH), Toronto, ON, Canada; Department of Psychology, University at Buffalo, SUNY, Buffalo, NY, USA

## Abstract

**Introduction:**

Dichotomized smoking abstinence (abstinent/smoking) is the standard outcome for clinical trials, but obscures smoking behavior change. Examining both smoking probability and smoking rate as outcomes may identify unique barriers to cessation (eg, craving, affect) among individuals who are receiving treatment but unable to quit.

**Aims and Methods:**

Two-part latent growth modeling examined daily smoking probability and smoking rate during the first week of the quit attempt using self-reported cigarettes per day. Smoking trajectories and the effect of 12 weeks of varenicline (vs. placebo), craving, negative affect (NA), and positive affect (PA) on these trajectories were examined among 828 adults in a randomized smoking cessation trial (NCT01314001).

**Results:**

On average, smoking probability was 46% on the target quit day and increased to 50% later into the week (*ps* < .01). Among participants continuing to smoke, daily smoking rates were reduced to 27% of baseline rates and remained stable throughout the week (*p* = .62). Varenicline use was associated with lower smoking probability (*p* < .001). Higher craving and NA were associated with a higher smoking probability and higher smoking rates (*p*s ≤ .05). Higher PA was associated with a higher smoking probability, but lower smoking rates (*p*s < .04).

**Conclusions:**

Modeling smoking behavior, versus dichotomized abstinence, reveals differences in predictors of treatment effects. Results suggest smoking probability increases early into the quit attempt while daily smoking rates remain stable. Varenicline increases the probability of abstinence. Smoking abstinence and lower smoking rates were both associated with lower cravings and NA. However, PA demonstrated differential relationships with abstinence and smoking rates.

**Implications:**

This study expands clinical outcomes beyond dichotomous smoking abstinence by conducting preliminary two-part latent growth models to evaluate treatment processes and barriers to cessation on both smoking probability and smoking rate. This approach provides a complementary understanding of treatment effects and predictors of outcomes. Results suggest time, treatment, craving, and affect may have differential relationships with smoking probability versus smoking rates which would not be captured in traditional modeling for clinical trial outcomes.

## Introduction

Over 34 million U.S. adults smoke cigarettes with about half reporting a past year quit attempt, but only 7%–8% report quitting.^1^ Though cessation yields the strongest health benefits, reducing cigarettes per day (CPD) improves the odds of abstinence in subsequent quit attempts.^2^ However, little research has evaluated treatment on daily smoking rates,^3,4^ and a call by *The Society for Research on Nicotine and Tobacco* advocates for clinical research to capture more detailed smoking outcomes.^5^ Traditionally, clinical trials dichotomize smoking status at one timepoint (eg, continuous abstinence at 6-month follow-up, 7-day point prevalence at end of treatment), overlooking nuanced information regarding smoking rates leading to conceptual challenges evaluating smoking behavior and methodological limitations (eg, reduced statistical power). Individuals who continue to smoke do so at varying rates, ranging from brief lapses to substantial reductions to a return to baseline smoking levels.^6^ Examining treatment effects and predictors of smoking rate versus cessation may inform interventions to lower daily smoking levels and re-initiate quit attempts.

Statistical techniques that simultaneously estimate the smoking probability and rates (ie, two-part latent growth modeling [LGM]) can inform how treatment uniquely affects these clinical outcomes.^7^ To date, no smoking cessation trials have used two-part LGMs to evaluate treatment effects and mechanisms on trajectories of smoking probability and rates. This study developed preliminary models to demonstrate the utility of two-part LGM via secondary analyses from a placebo-controlled varenicline trial.^5,8^ Varenicline provides an excellent initial test of this approach since it has shown efficacy for smoking abstinence and reducing smoking rates.^3^

Aim 1 characterized trajectories of smoking probability and smoking rates across the first week of the quit attempt. We focused on the first week since this is a critical window for lapse.^9,10^ Based on past research, the smoking probability was predicted to increase linearly across days,^11^ while smoking rates would either increase linearly or nonlinearly (eg, quadratic-rapid increases to stable smoking after a lapse).^6^ Aim 2 examined the effect of varenicline (vs. placebo) on trajectories of smoking probability and rates. It was predicted that participants in the varenicline group would have a lower probability of smoking and lower smoking rates based on prior clinical trials, including the parent trial for the current study.^3,8^ Additionally, work evaluating treatment mechanisms from the parent trial found that varenicline was associated with lower craving and negative affect (NA), which mediated binary smoking outcomes.^12^ However, it is unclear whether/how these processes separately impact day-level abstinence and smoking rates. Thus, Aim 3 tested whether higher craving and NA and lower positive affect (PA), were associated with higher smoking probability and smoking rates.

## Materials and Methods

### Participants and Procedure

Data were from a multi-site parent trial comparing smoking cessation treatments. Analyses included 828 adults randomly assigned to standard varenicline treatment or placebo. Inclusion criteria are described elsewhere (clinicaltrials.gov:NCT01314001).^8^ Procedures were approved by the institutional review board at each site and all participants provided informed consent. Of relevance to these analyses, participants answered questionnaires including timeline follow-back (TLFB) to assess the past week’s daily smoking at 1-week pre-quit (baseline), on the target quit day (TQD), and 1-week post-TQD. All participants were asked to abstain from smoking beginning the morning of their TQD.

### Measures

Smoking outcomes included self-reported smoking probability (binary daily smoking; yes/no) and percent of baseline smoking rate (continuous daily smoking) across the first week of the quit attempt. Percent of baseline smoking rate was calculated from TLFB as (cigarettes smoked that day/average baseline CPD) × 100 (eg, smoking an average of 10 CPD across the baseline week and reducing to 3 cigarettes on the TQD would equal a smoking rate of 30% for that day). Current craving was assessed using the Questionnaire on Smoking Urges-Brief^13^ and past week NA and PA were assessed using the Positive and NA Schedule.^14^

### Data Analysis

LGMs conducted in Mplus (version 8.10) evaluated trajectories of smoking probability and smoking rate over the first week of the quit attempt. Satorra-Bentler scaled chi-square difference tests compared model fit for nested tests with scaling corrections for non-normality. First, individual LGMs were estimated to determine the best-fitting trajectory for the slope of smoking probability and the slope of smoking rate.^15^ For smoking probability (binary), chi-square difference tests were used for nested comparisons of model log-likelihoods. For the smoking rate (continuous), MLR chi-square difference tests were used for nested model comparisons. Next, a two-part LGM simultaneously estimated smoking probability and rate on each of the first seven days of the quit attempt.^7^ The first “part” estimated day-to-day mean changes in smoking probability. Hypothetically, over the quit attempt, the average probability of smoking on the TQD maybe 50%, then increase to 55% on Day 1, a further increase to 60% on Day 2, etc. The second “part” estimated day-to-day mean changes in the smoking rate among those reporting smoking that day. Hypothetically, the average percent of baseline smoking rate maybe 20% on the TQD, then increase to 40% on Day 1 (smoking returning closer to baseline levels). Due to high collinearity with slope parameters, the intercept was assigned to Day 3 rather than Day 0 (TQD). Models separately evaluated the effect of treatment (Aim 2), craving, NA, and PA (Aim 3) on growth model parameters. Due to non-convergence, the quadratic slope term was removed for craving and PA models.

## Results

See Table S1 for participant characteristics.

### Aim 1: Trajectories of Smoking Probability and Rate

LGMs evaluated the fit of linear and quadratic slopes for smoking probability and smoking rate (Table S2). Nested chi-square tests indicated a random quadratic slope fit the data best for both smoking probability and smoking rate (*p*s < .001). Although there was significant individual variability in the quadratic slope for smoking rate (S^2^ = 0.50, *p* = .03), the mean estimate of the slope was nonsignificant (*m* = 0.01, *p* = .93). Further, estimation of the two-part LGM failed to converge with the inclusion of the quadratic slope for smoking rate. Since the linear slope model for smoking rate demonstrated an excellent fit and provided a more parsimonious solution, this model was used for the two-part LGM.

**Figure 1.**
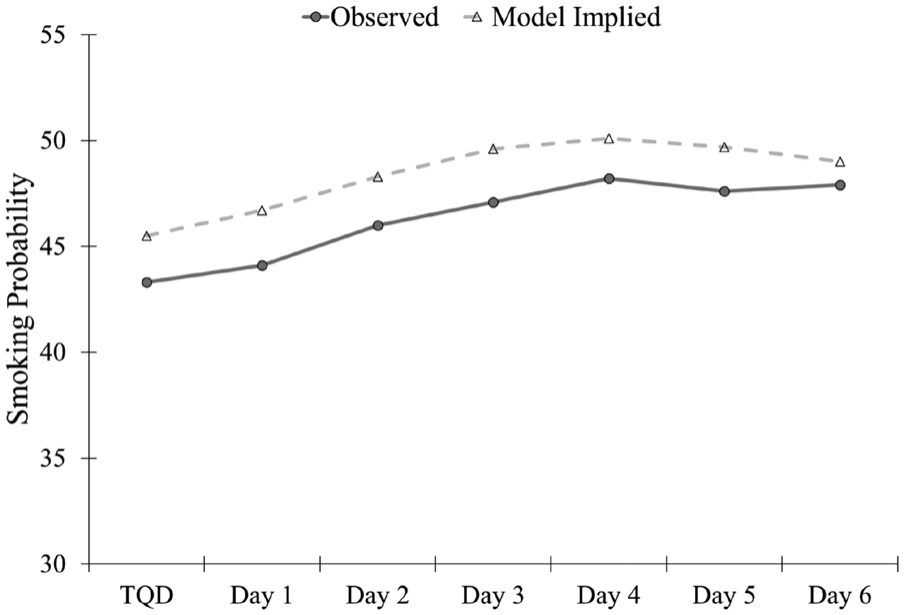
Observed and model implied mean smoking probability over the first week of the quit attempt. TQD = Target quit day.

**Figure 2.**
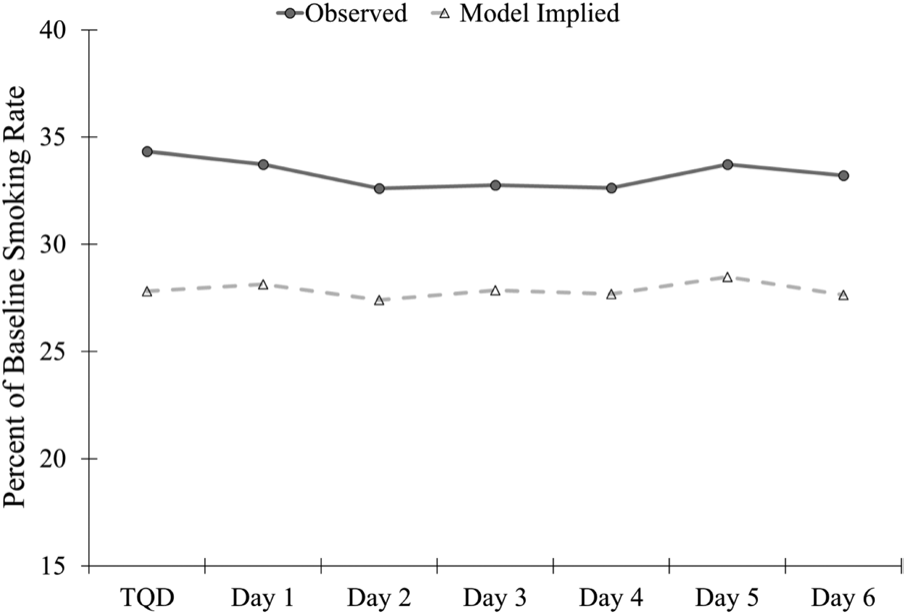
Observed and model implied percent of baseline smoking rate over the first week of the quit attempt. TQD = Target quit day.

The estimated probability of smoking was lowest on the TQD (46%), increased through Day 4 (50%; *m*_Linear_ = 0.13, SE = 0.05, *p* = .008), and remained stable through Day 6 (49%; *m*_Quadratic_ = −0.06, SE = 0.03, *p* = .01; Figure 1). There was significant individual variability in the intercept (S^2^ = 27.88, SE = 4.09, *p* < .001), linear (S^2^ = 0.55, SE = 0.13, *p* < .001), and quadratic (S^2^ = 0.19, SE = 0.08, *p* = .02) slope of smoking probability. The estimated mean percent of baseline smoking rate remained stable throughout the week ranging from 27% to 28% (*m*_Linear_ = 0.22, SE = 0.45, *p* = .62; Figure 2). There was significant individual variability in the intercept (S^2^ = 805.13, SE = 75.00, *p* < .001) and linear slope (S^2^ = 7.44, SE = 1.67, *p* < .001) of the smoking rate.

### Aim 2: Effect of Varenicline on Trajectories of Smoking Probability and Rate

Treatment with varenicline was associated with the intercept (Day 3) of smoking probability (*b* = −1.88, SE = 0.46, *p* < .001) and smoking rate (*b* = −5.59, SE = 2.34, *p* = .02; Table S3). The estimated smoking probability was lower in the varenicline group (42%) compared to the placebo group (54%). Smoking rates were similar between the varenicline group (27%) and the placebo group (29%). Treatment was not significantly related to the slopes of smoking probability or smoking rate (*p*s > .28).

### Aim 3: Effects of Craving and Affect on Trajectories of Smoking Probability and Rate

#### Craving

Higher pre-quit craving predicted a slightly less steep increase in the probability of smoking across the week (*b*_Linear_ = −0.06, SE = 0.03, *p* = .051), while higher TQD craving predicted a higher probability of smoking on Day 3 (*b*_Intercept_ = 0.63, SE = 0.19, *p* = .001) and higher smoking rates at Day 3 (*b*_Intercept_ = 3.24, SE = 0.94, *p* = .001).

#### NA

Higher pre-quit NA marginally predicted higher smoking probability on Day 3 (*b*_Intercept_ = 1.15, SE = 0.60, *p* = .054), while higher TQD NA predicted higher smoking rates on Day 3 (*b*_Intercept_ = 5.55, SE = 2.70, *p* = .04) and an acceleration in the increasing probability of smoking across the week (*b*_Quadratic_ = 0.19, SE = 0.05, *p* < .001).

#### PA

Higher pre-quit PA predicted a steeper increase in the probability of smoking across the week (*b*_Linear_ = 0.11, SE = 0.05, *p* = .03), while higher TQD PA predicted lower smoking rates at Day 3 (*b*_Intercept_ = −2.91, SE = 1.41, *p* = .04) and a less steep increase in the probability of smoking across the week (*b*_Linear_ = −0.13, SE = 0.05, *p* = .01).

Additional models examined the effect of growth model parameters on NA and PA at 1 week post quit (Section S1).

## Discussion

This study addresses the call to expand the assessment of clinical outcomes in tobacco cessation clinical trials beyond dichotomous smoking abstinence^5^ by conducting preliminary two-part LGMs to evaluate treatment effects and predictors of outcomes on both smoking probability and smoking rate. Less than half of the sample (46%) self-reported smoking on the TQD, which increased and then stabilized around 50% by the middle of the week. Among those who continued smoking, CPD reduced to approximately a quarter of baseline rates and remained stable over the week. While light use (1–4 CPD) is related to smoking-associated illnesses, there is a dose–response relationship between smoking rate and health outcomes.^16^ Further, light smoking is associated with future cessation; therefore, reduction may be an important step towards abstinence.^2^

This study provides novel insights into smoking occurring early into the quit attempt. Using 7-day point prevalence abstinence at 1-week post-quit, this study found the majority of participants lapsed (69% for the varenicline and 84% placebo group, respectively). However, the present results detail that day-to-day nearly half of participants self-reported abstinence and, among those reporting smoking, CPD was greatly reduced. These data suggest day-level factors, including craving and affect, influence short-term abstinence and reduced smoking rate, which may inform strategies for re-initiating abstinence (eg, identification/avoidance of high craving contexts).

Consistent with prior work,^3^ varenicline treatment was associated with a higher probability of smoking abstinence; however, smoking rates were similar between the treatment groups. Further, varenicline did not significantly influence trajectories of smoking behavior over the first week of the quit attempt. Effects may have been attenuated since smoking was assessed retrospectively and was not bio-verified. Varenicline has been shown to increase time to first lapse,^17^ but relying on self-reported smoking may have attenuated treatment effects on smoking probability trajectories within this study. Future work will face challenges with bioverification (eg, carbon monoxide) since most are not suitable for evaluating differences in the smoking rate, although novel methods (eg, nicotine metabolite panels) may have potential.^18^

Focusing on the first week of the quit attempt may have further obfuscated changes in smoking behavior and treatment effects. While early lapses are predictive of relapse, time to relapse after a lapse event is variable with relapse risk peaking 5–10 days post-TQD.^19^ Longer time intervals would allow for the identification of high lapse risk periods and variables associated with long-term maintenance of reduced smoking rates. Although one solution is to include more timepoints, increased computational demand may prevent model estimation, especially when multiple predictors and/or nonlinear changes are included.^20^ Model development decisions (eg, timeframes, predictors) should occur a priori. Although there was little change in smoking trajectories, there was significant variability, suggesting key individual differences may contribute to changes in smoking behavior. Future work utilizing this approach can evaluate participant factors (eg, cigarette availability, cue exposure) that contribute to differences in smoking trajectories.

In addition to treatment effects, common mediators of varenicline efficacy^12^ predicted smoking probability and smoking rate. Higher pre-quit craving was associated with less change in smoking probability, while those experiencing higher cravings on the TQD were more likely to smoke *and* report higher smoking rates. Individuals experiencing a consistently high craving, both pre- and post-quit, may have been more likely to maintain daily smoking, resulting in less change in smoking probability, higher smoking probabilities, and higher smoking rates. The increasing probability of smoking throughout the week was accelerated with higher NA and lower PA, suggesting a more rapid return to daily smoking. Further, those experiencing more NA and less PA on the TQD reported higher smoking rates. Unexpectedly, higher PA prior to the quit attempt was associated with increased smoking probability over time. Prior work suggests situations associated with higher PA (eg, social alcohol use) contribute to relapse and being in these situations more often (across pre- and post-quit) may increase the likelihood of smoking.^21^ However, elucidating such daily dynamics requires frequent assessments (eg, ecological momentary assessment) and momentary modeling (eg, dynamic structural equation models).

This work demonstrates the utility of two-part LGM to examine daily smoking probability and rates within a smoking cessation clinical trial and provides a preliminary evaluation of the effects of varenicline on smoking abstinence and rate following the call by *The Society for Research on Nicotine and Tobacco*.^5^ Varenicline reduced the probability of smoking but was largely unrelated to smoking rates and daily changes in smoking behavior early into the quit attempt. Craving and affect may be barriers to both smoking abstinence and rate. Future work can leverage information about barriers to smoking abstinence and reduction to improve outcomes.

## Supplementary Material

ntaf095_suppl_Supplementary_Material

## Data Availability

The data underlying this article were provided by the Pharmacogenetics of Nicotine Addiction Treatment Research Group by permission. Data will be shared on request to the corresponding author with the permission of the Pharmacogenetics of Nicotine Addiction Treatment Research Group.
