# Supplementary material for: Daily Cigarette Abstinence and Smoking Rate With Varenicline: Relationships With Treatment, Craving, and Affect During the First Week of the Quit Attempt
Source: Nicotine Tob Res. 2025 May 4;27(12):2328–32. doi: 10.1093/ntr/ntaf095 (PMC12641181; doi:10.1093/ntr/ntaf095)
Supplement: ntaf095_suppl_Supplementary_Material [file ntaf095_suppl_supplementary_material.docx]

| **Mean (SD; range)** | **Varenicline**  ***n = 420*** | **Placebo**  ***n = 408*** |
| --- | --- | --- |
| Age | 44.95 (11.71; 46) | 45.73 (11.07; 46) |
| Mean Baseline CPD | 16.73 (6.00; 42.57) | 17.31 (7.68; 67.14) |
| FTND | 5.10 (2.01; 10) | 5.36 (1.96; 10) |
| **Percent** |  |  |
| Female | 44.52 | 42.65 |
| Minoritized Race/Ethnicity | 43.57 | 41.91 |
| **Race *n*** |  |  |
| White/Caucasian | 232 | 225 |
| Black/African American | 160 | 151 |
| Asian | 14 | 13 |
| Other | 8 | 7 |
| Multi-racial | 5 | 12 |
| American Indian | 1 | 0 |
| **Ethnicity *n*** |  |  |
| Non-Hispanic/Non-Latinx | 398 | 382 |
| Hispanic/Latinx | 19 | 25 |
| Missing/Did not disclose | 3 | 1 |

**Supplemental Table 1.** Participant characteristics and smoking information.

**Note:** Treatment groups did not significantly differ on these variables (*p*s > .06). SD = Standard deviation. CPD = Cigarettes per day. FTND = Fagerström Test for Nicotine Dependence.

**Supplemental Table 2.** Nested model comparisons for the trajectories of smoking probability and smoking rate over the first week of the quit attempt.

| Individual Unconditional Latent Growth Model | | | |
| --- | --- | --- | --- |
| Smoking Probability: Nested Tests | | | |
|  | *Estimate (df)* | *p-value* | |
| *LL ∆ Intercept Only v. Linear Slope* | **99.69 (3)** | **< .001** | |
| *LL ∆ Linear Slope v. Quadratic Slope* | **43.95 (4)** | **< .001** | |
| Smoking Rate: Nested Tests | | | |
|  | *Estimate (df)* | | *p-value* |
| *χ^2^ ∆ Intercept Only v. Linear Slope* | **122.33 (3)** | | **< .001** |
| *χ^2^ ∆ Linear Slope v. Quadratic Slope* | **13.17 (4)** | | **.01** |
| Smoking Rate: Overall Model Fit | | | |
|  | *Estimate* | *Conclusion* | |
| *χ^2^ (df), p-value* | 35.00 (23), *p* = .052 | Good Fit | |
| *RMSEA* | 0.03 | Good Fit | |
| *CFI* | 0.99 | Good Fit | |
| *SRMR* | 0.03 | Good Fit | |

**Note:** Bolded values are statistically significant. All random intercepts and slopes. Binary outcome models do not produce overall model fit statistics, therefore, these estimates are unavailable for smoking probability models. df = Degrees of freedom. LL = Log-likelihood. RMSEA = Root mean square error of approximation. CFI = Comparative fit index. SRMR = Standardized root mean square residual.

| Unconditional Two-Part Latent Growth Model | | | | | |
| --- | --- | --- | --- | --- | --- |
|  | *Estimate (SE)* | | *p-value* | | |
| *Smoking Probability* |  | |  | | |
| *Threshold* | 0.06 (0.22) | | .78 | | |
| *Linear Slope* | **0.13 (0.05)** | | **.008** | | |
| *Quadratic Slope* | **-0.06 (0.03)** | | **.01** | | |
| *Smoking Rate* |  | |  | | |
| *Intercept* | **16.32 (1.34)** | | **< .001** | | |
| *Linear Slope* | 0.22 (0.45) | | .62 | | |
| Two-Part Latent Growth Model: Treatment Effects | | | | | |
| *Smoking Probability* | *Estimate (SE)* | | *p-value* | | |
| *Intercept* | **-1.88 (0.46)** | | **< .001** | | |
| *Linear Slope* | -0.11 (0.10) | | .28 | | |
| *Quadratic Slope* | -0.01 (0.05) | | .80 | | |
| *Smoking Rate* |  | |  | | |
| *Intercept* | **-5.95 (2.34)** | | **.02** | | |
| *Linear Slope* | 0.15 (0.34) | | .67 | | |
| Two-Part Latent Growth Model: Affect and Craving | | | | | |
|  | ***Pre-Quit Craving*** | | | ***TQD Craving*** | |
| *Smoking Probability* | *Estimate (SE)* | *p-value* | | *Estimate (SE)* | *p-value* |
| *Intercept* | 0.13 (0.17) | .44 | | **0.63 (0.19)** | **.001** |
| *Linear Slope* | **-0.06 (0.03)** | **.05** | | 0.05 (0.03) | .13 |
| *Quadratic Slope* | -- | -- | | -- | -- |
| *Smoking Rate* |  |  | |  |  |
| *Intercept* | 0.09 (0.74) | .90 | | **3.24 (0.94)** | **.001** |
| *Linear Slope* | -0.04 (0.09) | .68 | | -0.04 (0.11) | .71 |
|  | ***Pre-Quit Negative Affect*** | | | ***TQD Negative Affect*** | |
| *Smoking Probability* | *Estimate (SE)* | *p-value* | | *Estimate (SE)* | *p-value* |
| *Intercept* | **1.15 (0.60)** | **.05** | | -0.08 (0.51) | .88 |
| *Linear Slope* | 0.08 (0.14) | .57 | | -0.17 (0.13) | .18 |
| *Quadratic Slope* | -0.04 (0.07) | .58 | | **0.19 (0.05)** | **.001** |
| *Smoking Rate* |  |  | |  |  |
| *Intercept* | 3.06 (2.96) | .30 | | **5.55 (2.70)** | **.04** |
| *Linear Slope* | -0.59 (0.44) | .18 | | 0.22 (0.28) | .45 |
|  | ***Pre-Quit Positive Affect*** | | | ***TQD Positive Affect*** | |
| *Smoking Probability* | *Estimate (SE)* | *p-value* | | *Estimate (SE)* | *p-value* |
| *Intercept* | 0.40 (0.32) | .22 | | -0.44 (0.34) | .20 |
| *Linear Slope* | **0.11 (0.05)** | **.03** | | **-0.13 (0.05)** | **.01** |
| *Quadratic Slope* | -- | -- | | -- | -- |
| *Smoking Rate* |  |  | |  |  |
| *Intercept* | 2.23 (1.41) | .11 | | **-2.91 (1.41)** | **.04** |
| *Linear Slope* | -0.13 (0.18) | .47 | | 0.09 (0.17) | .57 |

**Supplemental Table 3.** Unconditional and conditional two-part latent growth model results.

**Note:** Bolded values are statistically significant. All random intercepts and slopes. Due to model non-convergence the quadratic slope term for smoking probability was removed for the craving and positive affect models. SE = Standard error.

**S1. SUPPLEMENTAL ANALYSES AND RESULTS**

**The relationship between affect 1 week post-quit on smoking probability and rate**

The PANAS was administered in this study to assess positive and negative affect over the past week. Due to the retrospective nature of this assessment, pre-quit and TQD PANAS scores were used to predict smoking probability and rate over the first week of the quit to establish temporal precedence in the primary analyses. However, PANAS scores collected at 1 week post-quit retrospectively evaluate affect over the first week of the quit. Although these concurrent reports prevent temporal precedence from being clearly established, PANAS scores from 1 week post-quit may be stronger predictors of smoking probability and rate during the first week of the quit attempt.

Aim 3 models examining the effect of affect at pre-quit and TQD on growth model parameters were re-ran to include NA and PA scores at 1 week post-quit. Consistent with the primary models, growth model parameters (outcome variables) were regressed on pre-quit and TQD NA and PA (predictor variables). However, since assessments of past week affect and cigarette use were concurrent at 1 week post-quit and to account for recency biases^1^ within the PANAS, 1 week post-quit NA and PA (outcome variables) were regressed on growth model parameters (predictor variables). Consistent with the primary models for PA, the quadratic slope term was removed from the model due to non-convergence.

Steeper increases in both the probability of smoking (*b*_Linear_=0.17, SE=0.07, *p*=.02) and smoking rate (*b*_Linear_=0.03, SE=0.01, *p*=.01) across the week were associated with higher NA at 1 week post-quit. No significant relationships were observed between growth model parameters and PA reported at 1 week post-quit (*p*s > .08). These results suggest retrospective reports of past week NA are associated with increasing risk of lapse and faster increases in daily smoking rates, returning closer to baseline CPD.

**SUPPLEMENTAL REFERENCES**

1. Stone A, Shiffman S, Atienza A, Nebeling L. *The science of real-time data capture: Self-reports in health research*. Oxford University Press; 2007.
